# Recent Advances in Nanoscale Zero-Valent Iron (nZVI)-Based Advanced Oxidation Processes (AOPs): Applications, Mechanisms, and Future Prospects

**DOI:** 10.3390/nano13212830

**Published:** 2023-10-25

**Authors:** Mingyue Liu, Yuyuan Ye, Linli Xu, Ting Gao, Aiguo Zhong, Zhenjun Song

**Affiliations:** 1 School of Pharmaceutical and Chemical Engineering, Taizhou University, Taizhou 318000, China; 2 Engineering Research Center of Recycling & Comprehensive Utilization of Pharmaceutical and Chemical Waste of Zhejiang Province, Taizhou University, Taizhou 318000, China

**Keywords:** nanoscale zero-valent iron (nZVI), advanced oxidation processes (AOPs), mechanisms, radical, reactive oxygen species (ROS), organic contaminants, degradation, recycle

## Abstract

The fast rise of organic pollution has posed severe health risks to human beings and toxic issues to ecosystems. Proper disposal toward these organic contaminants is significant to maintain a green and sustainable development. Among various techniques for environmental remediation, advanced oxidation processes (AOPs) can non-selectively oxidize and mineralize organic contaminants into CO_2_, H_2_O, and inorganic salts using free radicals that are generated from the activation of oxidants, such as persulfate, H_2_O_2_, O_2_, peracetic acid, periodate, percarbonate, etc., while the activation of oxidants using catalysts via Fenton-type reactions is crucial for the production of reactive oxygen species (ROS), i.e., •OH, •SO_4_^−^, •O_2_^−^, •O_3_CCH_3_, •O_2_CCH_3_, •IO_3_, •CO_3_^−^, and ^1^O_2_. Nanoscale zero-valent iron (nZVI), with a core of Fe^0^ that performs a sustained activation effect in AOPs by gradually releasing ferrous ions, has been demonstrated as a cost-effective, high reactivity, easy recovery, easy recycling, and environmentally friendly heterogeneous catalyst of AOPs. The combination of nZVI and AOPs, providing an appropriate way for the complete degradation of organic pollutants via indiscriminate oxidation of ROS, is emerging as an important technique for environmental remediation and has received considerable attention in the last decade. The following review comprises a short survey of the most recent reports in the applications of nZVI participating AOPs, their mechanisms, and future prospects. It contains six sections, an introduction into the theme, applications of persulfate, hydrogen peroxide, oxygen, and other oxidants-based AOPs catalyzed with nZVI, and conclusions about the reported research with perspectives for future developments. Elucidation of the applications and mechanisms of nZVI-based AOPs with various oxidants may not only pave the way to more affordable AOP protocols, but may also promote exploration and fabrication of more effective and sustainable nZVI materials applicable in practical applications.

## 1. Introduction

In the past few decades, the anthropogenic pollution of water bodies has become one of the most problematic crises facing the world [1,2,3]. Large quantities of pollutants, such as dyes, antibiotics, pesticides, chlorinated organics, polycyclic aromatic hydrocarbons, and personal care products, have been discharged into water, posing severe threats to ecosystems and human beings [4,5,6]. Therefore, the development of an efficient, eco-friendly, and cost-effective approach for the removal of industrial and municipal water pollutants, which have attracted extensive attention, is of utmost importance [7,8,9].

Various technologies have been developed for the remediation of wastewater, such as adsorption, reduction, dechlorination, biodegradation, and advanced oxidation processes (AOPs) [10,11,12]. Among these techniques, AOPs have received considerable attention in the last decade due to their mild reaction conditions (room temperature and ambient pressure), high efficiency, and extensive adaptability in decontamination [13,14]. In an advanced oxidation process (AOP), organic pollutants, including refractory, persistent and toxic organic pollutants, can be non-selectively oxidized and mineralized into CO_2_, H_2_O, and inorganic salts using reactive oxygen species (ROS). These free radicals, which are generated from various oxidants, such as persulfate, hydrogen peroxide (H_2_O_2_), oxygen (O_2_), peracetic acid, periodate, and percarbonate, have a strong oxidation capability and high reaction efficiency with regard to the elimination of organic pollutants [15,16,17]. AOPs can be regarded as a versatile and reliable technology for environmental remediation, owing this to their availability to provide various approaches to produce ROS [18,19,20]. The hydroxyl radical (•OH) and sulfate radical (•SO_4_^−^) are the most common free radicals being generated in AOPs [21,22]. The high redox potentials of •SO_4_^−^ (E^0^ = 2.5–3.1 V) and •OH (E^0^ = 1.9–2.7 V) provide AOPs with a significant advantage in the degradation of recalcitrant organic pollutants [23,24]. Other ROS may also be generated in AOPs which are conducted using nZVI, i.e., •O_2_^−^, •O_3_CCH_3_, •O_2_CCH_3_, •IO_3_, •CO_3_^−^, and ^1^O_2_ [25,26].

**Scheme 1:**
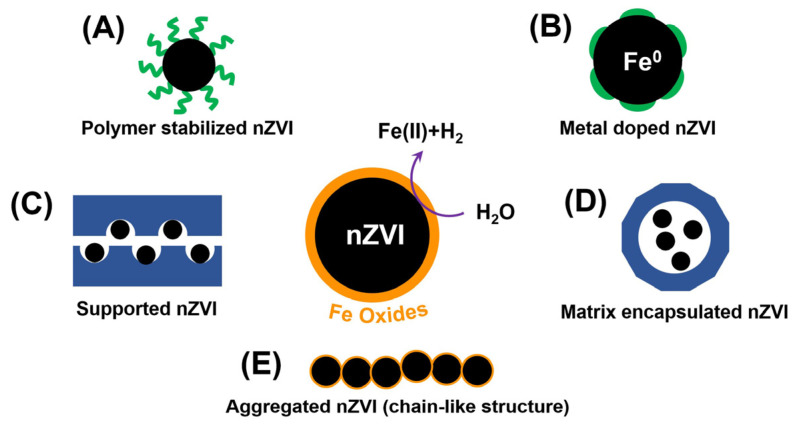
Scheme of the oxidation of nZVI in solution and the structures of nZVI materials. (**A**) Polymer-stabilized nZVI; (**B**) metal-doped nZVI; (**C**) porous material-supported nZVI; (**D**) matrix-encapsulated nZVI; (**E**) chain-like structure of aggregated nZVI.

The activation method of oxidants is crucial for the production of high oxidative ROS [27]. The activation methods generally include physical activation, chemical activation, and catalytic activation, the former of which needs exterior energy (heat, ultrasonic, or ultraviolet) for the generation of radicals, leading to some inevitable disadvantages comprising harsh reaction conditions, a high energy requirement, and complicated operation and equipment [28,29]. The chemical activation method, adopting an alkaline metal, phenol, or quinone as the activator, not only requires a high dosage of these chemicals, but can also produce toxic substances [30,31]. Compared with the above-mentioned two activation methods, catalytic activation with Cu, Co, Ce, Mn, or Fe-based catalytic materials is recognized as one of the most effective and easy-to-operate activation methods in AOPs [32,33]. Moreover, some of these catalysts can release toxic metal ions into the water and lead to the risk of secondary pollution, imposing constraints on their practical applications [34,35]. As iron is earth abundant (second most abundant metallic element), cheap, and environmentally friendly, Fe-based catalysts have been considered as one of the best-performing catalysts in AOPs, in which Fe^2+^ is generally recognized as the main effective activator. Unfortunately, soluble ferrous salt not only generates undesirable iron sludge during the degradation process, but it can also consume the generated radicals by acting as the scavenger when there is an excess of ferrous ions [36,37]. As an alternative, nZVI has a core consisting of Fe^0^ and can gradually release ferrous ions, providing a sustained activation effect in AOPs [38,39]. nZVI has a high reducibility and reactivity, and can be oxidized with H_2_O to generate Fe(II) and H_2_ (Scheme 1) [40]. The oxidation of nZVI with H_2_O and dissolved oxygen will lead to the formation of an oxide layer (Fe_x_O_y_) on nZVI, resulting in a common core–shell structure of an Fe nanoparticle (NP) (Scheme 1) [41]. The dispersed nZVI in the solution tends to aggregate and form chain-like aggregations due to magnetic interactions and van der Waals forces, thus reducing the specific surface area and the reactivity of nZVI [42,43]. Therefore, to obtain a high reactive and stable nZVI material, nZVI must be stabilized with a polymer, doped with another metal, supported by a porous material, or encapsulated in a matrix (Scheme 1). nZVI can be easily synthesized via the reduction of ferric salt wit borohydride, during which stabilizers or supports are frequently employed to suppress the aggregation of Fe nanoparticles (NPs) [44,45]. With a particle size of less than 100 nm in diameter, nZVI has a high reducibility and adsorption capacity [46]. Additionally, nZVI is cheap, highly reactivity, easily recoverable, and can be easily recycled with an external magnet, and has a low impact on the environment, necessitating it as a promising heterogeneous AOP catalyst [47,48].

Therefore, nZVI has garnered great attention in environmental remediation issues in the past decade [49,50]. With its outstanding performance in terms of the removal of contaminants, nZVI has been applied in the remediation of chlorinated organics, nitroaromatics, dyes, nitrate, phosphate, and heavy metals in wastewater and contaminated soils [51,52]. To help the readers get a complete framework on the application of nZVI in AOPs and plan their further research on this topic, it must be explained that there is an abundance of intensive research studies on the degradation of organic pollutants using the combination of AOPs with nZVI materials; however, there is a lack of papers that critically analyze the advances in the applications of nZVI in AOPs and relating mechanisms, especially in the comprehensive examination of the employment of nZVI in the activation of different oxidants,. In this regard, this review aims to provide an overview of the recent advances of the applications, mechanisms, and prospects of nZVI-catalyzed AOPs with persulfate, H_2_O_2_, O_2_, peracetic acid, periodate, and percarbonate as oxidants.

## 2. Persulfate-Based AOPs

Peroxymonosulfate (SO_5_^2−^, PMS) and peroxydisulfate (S_2_O_8_^2−^, PDS) are two common persulfates that are utilized in AOPs, both of which are chemically stable under mild conditions when they are not activated [53,54]. Taking advantage of easy separation, heterogeneous activation has attracted increasing attention compared with homogeneous systems [55], especially for nZVI, which can be conveniently recovered by a magnet and recycled. When activated with nZVI, the hydro-peroxide bond (O−O) of persulfate can be broken via homolytic or heterolytic cleavage and can generate various ROS for the degradation of organic pollutants [34].

### 2.1. Applications

The applications of persulfate-based AOPs are summarized in Table 1. nZVI and the nZVI supported by porous materials were adopted for the activation of persulfate (Na_2_S_2_O_8_, K_2_S_2_O_8_, or KHSO_5_) for the degradation of organic pollutants, such as chlorophenol (CP), phenols, dichlorophenol (DCP), sulfamethazine (SMZ), sulfamethoxazole (SMX), atrazine, oxytetracycline (OTC), trichloroethylene (TCE), tetracycline (TC), tetrabromobisphenol A (TBBPA), methyl orange (MO), bisphenol A (BPA), organophosphorus pesticides (OPPs), pyrene, rhodamine B (RhB), gamma-hexachlorocyclohexane (γ-HCH), and various antibiotics (Table 1).

**Figure 1:**
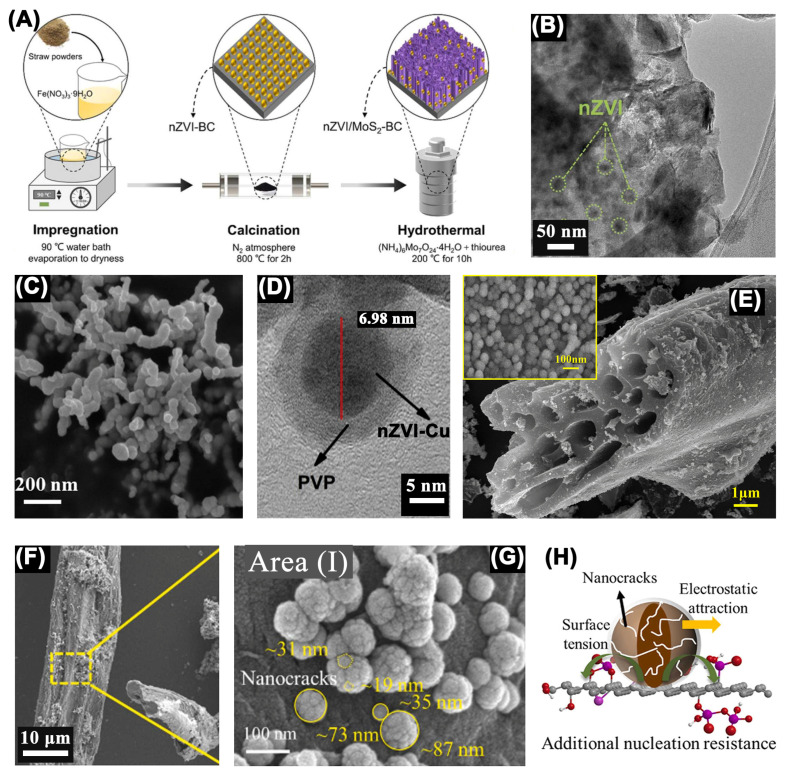
(**A**) The diagram of the nZVI/MoS_2_-BC preparation; (**B**) TEM images of nZVI/MoS_2_-BC at 50 nm [84], Copyright 2023 Elsevier. (**C**) SEM image of nZVI/Co_3_O_4_ [60], Copyright 2020 Elsevier. (**D**) TEM image of PVP-nZVI-Cu [71], Copyright 2021 Elsevier. (**E**) SEM image of nZVI/CuO@BC, the insert comprises nZVI particles with diameter of ~50 nm that are uniformly dispersed on the surface and in the tubes of the CuO/BC composites [73], Copyright 2021 Elsevier. (**F**) SEM image of nZVI@P-BC; (**G**) SEM image of nanocracks on nZVI nanosphere surface; (**H**) the formation mechanism of nanocracked nZVI [85], Copyright 2023 Elsevier.

The catalytic application of nZVI in environmental remediation can be seriously hindered with agglomeration and passivation that arises from the high surface energy and high surface activity of NPs [89]; more and more studies have loaded nZVI on/in a porous support, such as carbon, reduced graphene oxide (rGO), reduced graphene oxide aerogel (rGOA), biochar (BC), cotton carbon fiber (CF), graphene-like carbon sheet (CS), MoS_2_ nanosheets, and so on [90]. Supported and polymer-modified nZVI was usually spherical [71,73,84]; however, special geometric forms of nZVI, such chains or nanocracked spheres (Figure 1), may also be formed due to magnetic interactions, van der Waals forces, or the regulation of soluble functional species (i.e., phosphorus species, polyphenols) [45,60,85,91].

**Figure 2:**
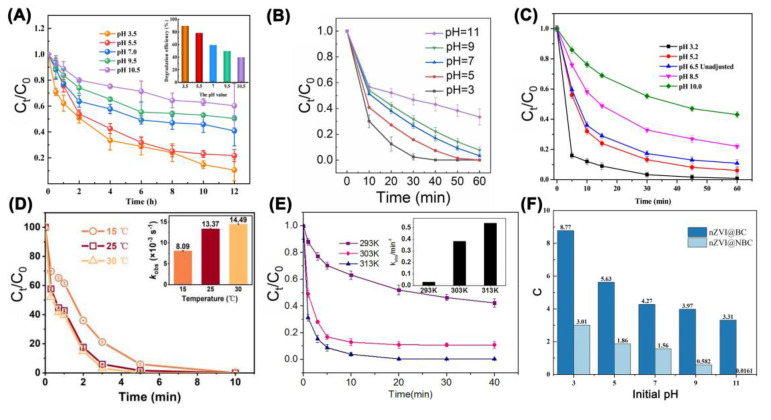
(**A**) Effects of initial pH on TBBPA degradation efficiency [93], Copyright 2021 Elsevier. (**B**) Influences of pH conditions on RhB degradation [84], Copyright 2023 Elsevier. (**C**) Effects of solution pH on TCE removal in PVP-nZVI-Cu system [71], Copyright 2021 Elsevier. (**D**) Degradation of atrazine with nZVI-Cu^0^ at various temperatures [40], Copyright 2023 Elsevier. (**E**) Effects of temperature on the degradation of norfloxacin [62], Copyright 2020 Elsevier. (**F**) The Fe leaching of nZVI@NBC system and nZVI@BC system during the reaction under different initial pH values [77], Copyright 2022 Elsevier.

**Figure 3:**
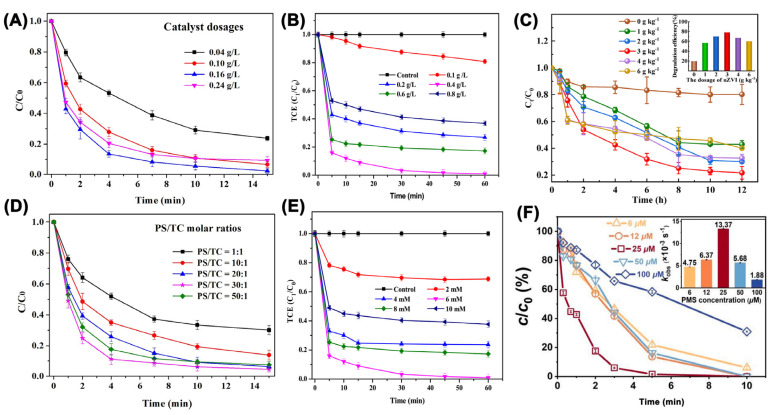
(**A**) Effects of catalyst dosage on TC catalytic degradation [60], Copyright 2020 Elsevier. (**B**) Effects of PVP-nZVI-Cu dosage on TCE removal [71], Copyright 2021 Elsevier. (**C**) Effects of different dosages of nZVI on degradation efficiency of TBBPA [93], Copyright 2021 Elsevier. (**D**) Effects of different persulfate concentrations on TC catalytic degradation [60], Copyright 2020 Elsevier. (**E**) Effects of different persulfate concentrations on TCE removal [71], Copyright 2021 Elsevier. (**F**) Effects of different persulfate concentration on atrazine catalytic degradation using nZVI-Cu^0^ [40], Copyright 2023 Elsevier.

**Figure 4:**
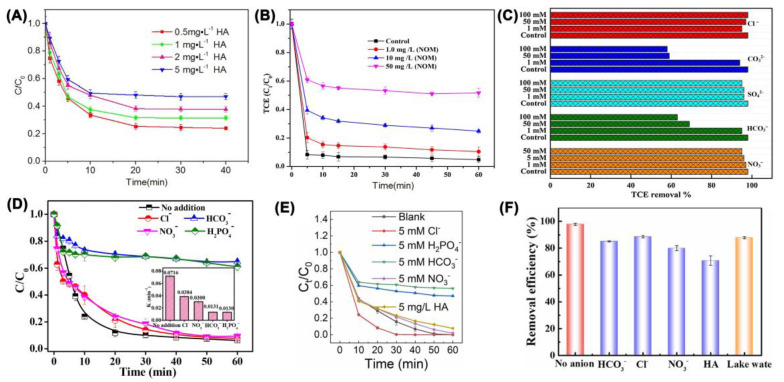
(**A**) Effects of humic acid (HA) on the degradation of norfloxacin [62], Copyright 2020 Elsevier. (**B**) Effects of natural organic matter (NOM) and (**C**) inorganic ions on TCE degradation in nZVI-Ni@BC-persulfate system [72], Copyright 2021 Elsevier. (**D**) Levofloxacin degradation efficiency under different inorganic anions [69], Copyright 2021 Elsevier. (**E**) Influencec of different anions on RhB degradation [84], Copyright 2023 Elsevier. (**F**) Effects of co-existing anions and organic matter on BPA treatment performance with Fe^0^-CNTs catalytic membrane system [94], Copyright 2022 Elsevier.

The degradation efficiency of the organic pollutants depends on multiple factors, such as temperature, pH of the reaction solution, catalyst dosage, persulfate concentration, and impurities in water [92]. In general, an increased temperature and a low pH were classified as favorable conditions for the removal of pollutants (Figure 2); however, they are inhibited when there is an excess of nZVI or persulfate (Figure 3), considering the rapid self-scavenging ability of un-reacted ROS [68,81]. The presence of natural organic compounds and inorganic anions in the environment was an unfavorable condition for the removal of pollutants (Figure 4) [62,78].

**Figure 5:**
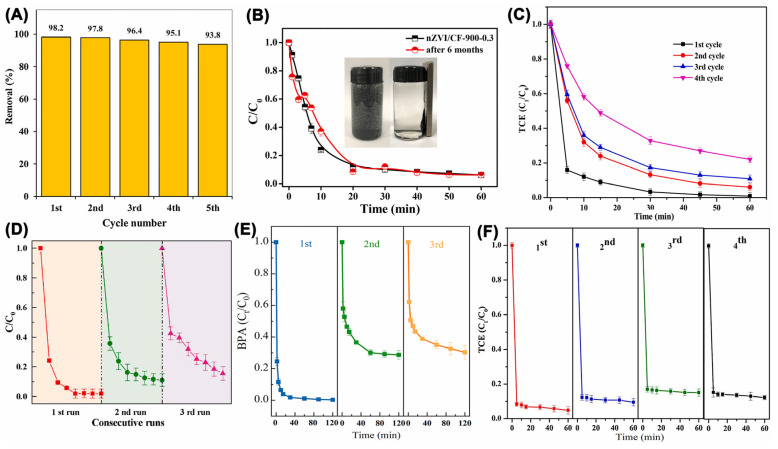
(**A**) Reusability of nZVI-rGO under five consecutive oxidation cycles [58], Copyright 2020 Elsevier. (**B**) Levofloxacin removal efficiency of nZVI/CF after 6 months (inset: the magnetic separation of the nZVI/CF dispersed in levofloxacin) [69], Copyright 2021 Elsevier. (**C**) TCE removal in different cycles using PVP-ZVI-Cu nanoparticles in persulfate environment [71], Copyright 2021 Elsevier. (**D**) Reusability study of nZVI-BC [87], Copyright 2023 Elsevier. (**E**) Reusability of nZVI@NBC/persulfate system [77], Copyright 2022 Elsevier. (**F**) Stability and durability of nZVI-Ni@BC catalyst [72], Copyright 2021 Elsevier.

Notably, these research studies revealed that nZVI materials can be conveniently recovered by a magnet and reused 3–5 times in AOPs (Figure 5), which reinforces their potential recyclable application in practice [58,69,71,72,77,87]. After storage for 6 months, nZVI/CF still had a favorable levofloxacin degradation efficiency, which confirms that nZVI materials can possess excellent long-term stability (Figure 5B) [69], while some recycling experiments in the reported research suffered an obvious decrease in degradation efficiency [60,79,88], for example, the degradation rate of atrazine was only 40.1% in the seconds run, which is a significant reduction of 53.7% below the initial rate [70].

Organic pollutants not only exist in water systems, they can also be adsorbed and accumulated in soil systems, causing soil pollution and adverse effects on the growth of crops [93]. Therefore, there is an important theoretical and practical significance for the development of efficacious remediation technologies for the degradation of organic pollutants in soil [95,96]. Compared with the methods of biological degradation, physical adsorption, and reduction for the remediation of polluted soil, AOPs have more advantages concerning efficiency and operational costs [93,97]. Significantly, persulfate-based AOPs conducted using nZVI has been applied for the remediation of TBBPA-polluted soil (concentration of TBBPA was 5 mg/kg soil, nZVI dosage was 3 g/kg soil, and reaction temperature was 25 °C), petroleum-polluted soil (concentration of total petroleum hydrocarbons was 6625 ± 115 mg/kg soil, nZVI dosage was 2 g/kg soil, and reaction temperature was 25 °C), and anthracene-polluted soil (concentration of anthracene was 100 mg/kg soil, nZVI dosage was 1.77 g/kg soil, and reaction temperature was 20 °C), yielding a removing efficiency of 78.32% in 12 h, 96% in 10 h, and 76.4% in 12 h, respectively [56,66,93,97].

In addition, some recent investigations indicated that nZVI-based AOP treatment has the potential to destroy antibiotic resistance genes that are released from damaged antibiotic-resistant bacteria in sewage and sludge, which may be beneficial to the enhancement of the performance of disinfection and the alleviation of bacterial resistance risks [98,99]. Specifically, Duan et al. adopted *Ginkgo biloba* L. leaf extract-modified nZVI to activate PS to produce ROS for the removal of antibiotic resistance genes, and achieved satisfactory removal efficiencies towards *sul*1, *intI*1, and the bacterial 16S rRNA gene [99,100].

### 2.2. Mechanisms

The activation mechanism of persulfate using nZVI relies on the corrosion of the core of nZVI and the resultant release of ferrous ions [101]. The possible generation pathway of Fe^2+^ in persulfate-based AOPs conducted using nZVI includes the oxidation of nZVI with water, oxygen, and persulfate, which is described as Equations (1)–(4). After the formation of Fe^2+^, •SO_4_^−^ is subsequently produced via Equation (5). The presence •SO_4_^−^ radicals can also cause a reaction with H_2_O and OH^−^ that generates •OH (Equations (6) and (7)) [72]. Moreover, persulfate (besides being able to be activated with Fe^2+^) can also be directly activated with Fe^0^ via electron transfer to generate •SO_4_^−^ and •OH (Equations (8) and (9)), both of which are the major ROS generating in persulfate-based AOPs and which played a predominant role in the oxidized degradation of organic pollutants [93]. However, excessive •SO_4_^−^ or Fe^2+^ can result in a rapid elimination of •SO_4_^−^ (Equations (10) and (11)), diminishing the removal efficiency of the pollutants [87,93]. The byproduced Fe^3+^ can be further reduced with nZVI and regenerate Fe^2+^ (Equation (12)), yielding an Fe^2+^/Fe^3+^ cycle, and thus providing nZVI with persistent reactivity for the activation of persulfate [40,65,87].

Fe^0^ + 2H_2_O → Fe^2+^ + H_2_ ↑ + 2OH^−^ (1)

2Fe^0^ + O_2_ + 2H_2_O → 2Fe^2+^ + 4OH^−^ (2)

Fe^0^ + O_2_ + 2H^+^ → Fe^2+^ + H_2_O_2_ (3)

Fe^0^ + S_2_O_8_^2−^ → Fe^2+^ + 2SO_4_^2−^ (4)

Fe^2+^ + S_2_O_8_^2−^ → Fe^3+^ + SO_4_^2−^ + •SO_4_^−^ (5)

•SO_4_^−^ + OH^−^ → SO_4_^2−^ + •OH (6)

•SO_4_^−^ + H_2_O → SO_4_^2−^ + •OH + H^+^ (7)

Fe^0^ + 2S_2_O_8_^2−^ → Fe^2+^ + SO_4_^2−^ + •SO_4_^−^ (8)

Fe^0^ + 2S_2_O_8_^2−^ + 2H_2_O → Fe^2+^ + 4SO_4_^2−^ + 2•OH + 2H^+^ (9)

•SO_4_^−^ + Fe^2+^ → SO_4_^2−^ + Fe^3+^ (10)

•SO_4_^−^ + •SO_4_^−^ → 2SO_4_^2−^ or S_2_O_8_^2−^ (11)

2Fe^3+^ + Fe^0^ → 3Fe^2+^ (12)

Furthermore, supposing that the persulfate is PMS, the formation pathway of •OH has a slight difference; that is, Fe^2+^ catalyzes HSO_5_^−^ to produce •OH or •SO_4_^−^, as shown in Equations (13) and (14) [40,69,83,88]. PMS can also directly react with Fe^0^ via electron transfer to generate •SO_4_^−^ (Equation (15)), similarly to the activation mechanism of PDS [84].

Fe^2+^ + HSO_5_^−^ → Fe^3+^ + OH^−^ + •SO_4_^−^ (13)

Fe^2+^ + HSO_5_^−^ → Fe^3+^ + •OH + SO_4_^2−^ (14)

Fe^0^ + 2HSO_5_^−^ → Fe^2+^ + 2OH^−^ + 2•SO_4_^−^ (15)

**Figure 6:**
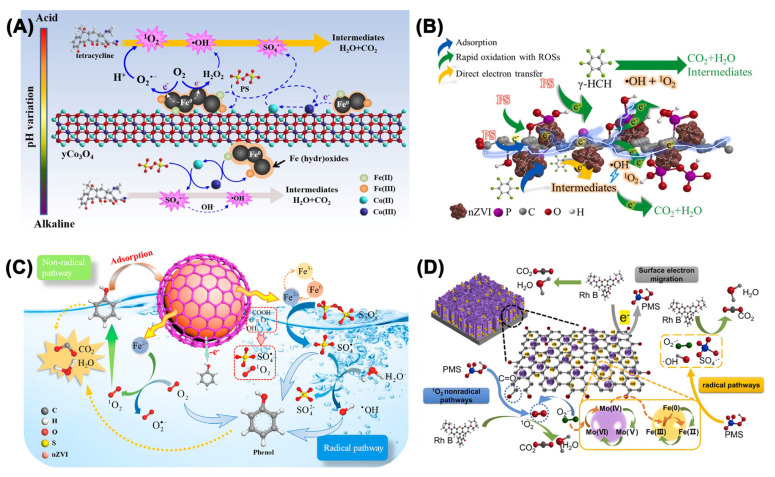
(**A**) Proposed ROS evolution mechanisms in nZVI/yCo_3_O_4_/persulfate system [60], Copyright 2020 Elsevier. (**B**) Degradation mechanisms of γ-HCH in the nZVI@P-BC/persulfate system [85], Copyright 2023 Elsevier. (**C**) Mechanism scheme for phenol degradation in nZVI-BC/persulfate system [87], Copyright 2023 Elsevier. (**D**) Proposed mechanisms of radical pathways and nonradical pathways in nZVI/MoS_2_-BC/persulfate system [84], Copyright 2023 Elsevier.

According to the reported studies on persulfate-based AOPs, both •SO_4_^−^ and •OH are considered as the major ROS that are responsible for the degradation of organic contaminants (Table 1) [15,64,65,67,71,72,73,74,75,76,81,82]. With regard to the dominant ROS, a few research studies revealed that •OH played a dominant role in the degradation compared to •SO_4_^−^. This may be attributed to the hydrolysis of SO_4_^−^ (Equations (6) and (7)) during the reaction, which can be promoted by the modification of nZVI, and thus generate more •OH and incidentally cause the system to be acidic [57,63]. Additionally, most of the research stated that •SO_4_^−^ plays a more dominant role in the reaction compared to •OH [40,59,66,68,69,80,83,84,88]. Investigations suggested that an acidic environment is favorable for the generation of •SO_4_^−^, while neutral and alkaline conditions were more conducive to the formation of •OH (Figure 6A); hence, this could have affected the domination of •OH and •SO_4_^−^ in the degradation process [60,76]. The acidic condition is rich in H^+^ and scarce in OH^−^, both of which are adverse to the formation of •OH via Equations (6) and (7). Instead, the alkaline condition is rich in OH^−^ and scarce in H^+^, which will facilitate the reaction of Equations (6) and (7) and transform more •SO_4_^−^ ions into •OH.

Overall, acid condition is suitable for the activation of persulfate [57]. In fact, an acid condition is a benefit for the formation of Fe^2+^ via the reaction of Fe^0^ and H^+^ (Figure 2F); furthermore, the surface of a catalyst will be negative charged in the alkaline condition, which will cause the catalyst to repel the persulfate anion (HSO_5_^−^ or S_2_O_8_^2−^) and further inhibit the activation of persulfate [57]. Thus, the optimal pH of the reaction solution for the degradation of contaminants is usually acidic (Figure 2), for instance, 4-CP can degrade more effectively in a pH of 6 than in a pH of 11 [57].

Aside from the above-mentioned dominant ROS modes in persulfate-based AOPs conducted with nZVI, some researchers declared that the superoxide radical (•O_2_^−^) and singlet oxygen (^1^O_2_) may also be the dominant ROS for the degradation of contaminants (Table 1) [77,85,86,87]. Huang et al. prepared supported nZVI with P-doped biochar and employed it in persulfate-based AOPs, in which ^1^O_2_ and •OH were verified as the dominant ROS (Figure 6B) [85]. A study conducted by Cao et al. demonstrated that •O_2_^−^ and ^1^O_2_ played dominant roles in their AOP system (Figure 6C) [87]. The probable generation pathway of •O_2_^−^ and ^1^O_2_ is shown as Equations (16)–(21). The formation of •O_2_^−^ (E^0^ = 1.56 V) is originated from the hydrolysis of persulfate and the reaction between Fe^2+^ and O_2_ (Equations (16)–(18)) [102]. ^1^O_2_ (E^0^ = 0.81 V) is usually generated from the reactions among •O_2_^−^, •OH, and •SO_4_^−^ via Equations (19)–(21) [79,80]. And, the introduction of N species on the nZVI material is commonly considered to be a profit for the generation of ^1^O_2_ [77].

S_2_O_8_^2−^ + 2H_2_O → 2SO_4_^2−^ + HO_2_^−^ + 3H^+^ (16)

S_2_O_8_^2−^ + HO_2_^−^ → SO_4_^2−^ + •SO_4_^−^ + •O_2_^−^ + H^+^ (17)

Fe^2+^ + O_2_ → •O_2_^−^ + Fe^3+^ (18)

2•O_2_^−^ + 2H^+^ → ^1^O_2_ + H_2_O_2_ (19)

•O_2_^−^ + •OH → ^1^O_2_ + OH^−^ (20)

•O_2_^−^ + •SO_4_^−^ → SO_4_^2−^ + ^1^O_2_ (21)

In the research conducted by Huang et al., the activation mechanism of persulfate was proposed, as nonradicals (^1^O_2_) dominated the pattern in acidic conditions and radicals (•SO_4_^−^) dominated the pattern in alkaline conditions [60]. Besides the H^+^/OH^−^-dependent reaction pathway determines the formation of ROS (Equations (19) and (20)), the electron donating capacity of nZVI at different pH and the M^(n+1)+^/M^n+^ redox cycles between Fe species or doping metals (i.e., the Co metal) also matters [60]. Additionally, results from the electron paramagnetic resonance (EPR), X-ray photoelectron spectrum (XPS), and a series of screening experiments revealed the synergistic effect between Fe and Co/Cu/Ni/Mo/Mn (Figure 6D) in redox cycling (Equations (22)–(31)) [40,60,62,71,72,73,78,81,84].

Co^2+^ + S_2_O_8_^2−^ → Co^3+^ + SO_4_^2−^ + •SO_4_^−^ (22)

Fe^2+^ + Co^3+^ → Fe^3+^ + Co^2+^ (23)

Cu^+^ + S_2_O_8_^2−^ → Cu^2+^ + •SO_4_^−^ + SO_4_^2−^ (24)

2Cu^2+^ + Fe^0^ → 2Cu^+^ + Fe^2+^ (25)

Ni^+^ + S_2_O_8_^2−^ → Ni^2+^ + •SO_4_^−^ + SO_4_^2−^ (26)

2Ni^2+^ + Fe^0^ → 2Ni^+^ + Fe^2+^ (27)

Mo^4+^ + HSO_5_^−^ → Mo^5+^ + •SO_4_^−^ + OH^−^ (28)

Mo^5+^ + HSO_5_^−^ → Mo^6+^ + •SO_4_^−^ + OH^−^ (29)

Mo^4+^ + 2Fe^3+^ → Mo^6+^ + 2Fe^2+^ (30)

Mn^2+^ + HSO_5_^−^ → Mn^3+^ + SO_4_^2−^ + •OH (31)

Additionally, regulating the reactivity of nZVI alters the production rate of Fe^2+^, imposing an impact on the generation of ROS and further affecting the degradation efficiency of contaminants [75]. Furthermore, inorganic anions (Cl^−^, Br^−^, NO_3_^−^, CO_3_^2−^, HCO_3_^−^, PO_4_^3−^, H_2_PO_4_^−^) and natural organic matter (i.e., humic acid (HA)) existing ubiquitously in natural water can often bring varying diverse effects on the degradation efficiency of contaminants (Figure 4) [60,70,73,81,103]. This may attribute not merely to inorganic anions having the ability to quench ROS, such as •OH and •SO_4_^−^, and generating less oxidative capacity free radicals (Equations (32)–(37)), but to HA as well [104]. HA can not only competitively react with radicals, but can also block the active sites of catalyst, resulting in an increased inhibiting effect on the removal efficiency of contaminants with the increase of their concentration [71,73,81,87]. As for the intensity of the inhibitory effects, the results in the research conducted by Diao et al. indicated that the effect occurred as follows: HA > HCO_3_^−^ > PO_4_^3−^ > NO_3_^−^ > Cl^−^ [67]. Alternatively, results in the research conducted by Rao et al. indicated that it occurred as follows: HCO_3_^−^ > SO_4_^2−^ > Cl^−^ > NO_3_^−^ [73]. Furthermore, HCO_3_^−^ and H_2_PO_4_^−^ are believed to impose a more significant inhibiting effect on contaminant degradation compared with other inorganic anions (Figure 4), and owe this to the low oxidative capacity of their corresponding free radicals and their complex with Fe^2+^, the latter of which will make Fe species unavailable for persulfate activation [70,71,105].

Cl^−^ + •SO_4_^−^ → SO_4_^2−^ + •Cl Cl^−^ + •OH → OH^−^ + •Cl (32)

Br^−^ + •SO_4_^−^ → SO_4_^2−^ + •Br Br^−^ + •OH → OH^−^ + •Br (33)

NO_3_^−^ + •SO_4_^−^ → SO_4_^2−^ + •NO_3_ NO_3_^−^ + •OH → OH^−^ + •NO_3_ (34)

CO_3_^2−^ + •SO_4_^−^ → SO_4_^2−^ + •CO_3_^−^ CO_3_^2−^ + •OH → OH^−^ + •CO_3_^−^ (35)

HCO_3_^−^ + •SO_4_^−^ → SO_4_^2−^ + •HCO_3_ HCO_3_^−^ + •SO_4_^−^ → SO_4_^2−^ + •CO_3_^−^ + H^+^ (36)

HCO_3_^−^ + •OH → OH^−^ + •HCO_3_ HCO_3_^−^ + •OH → H_2_O + •CO_3_^−^ (37)

**Figure 7:**
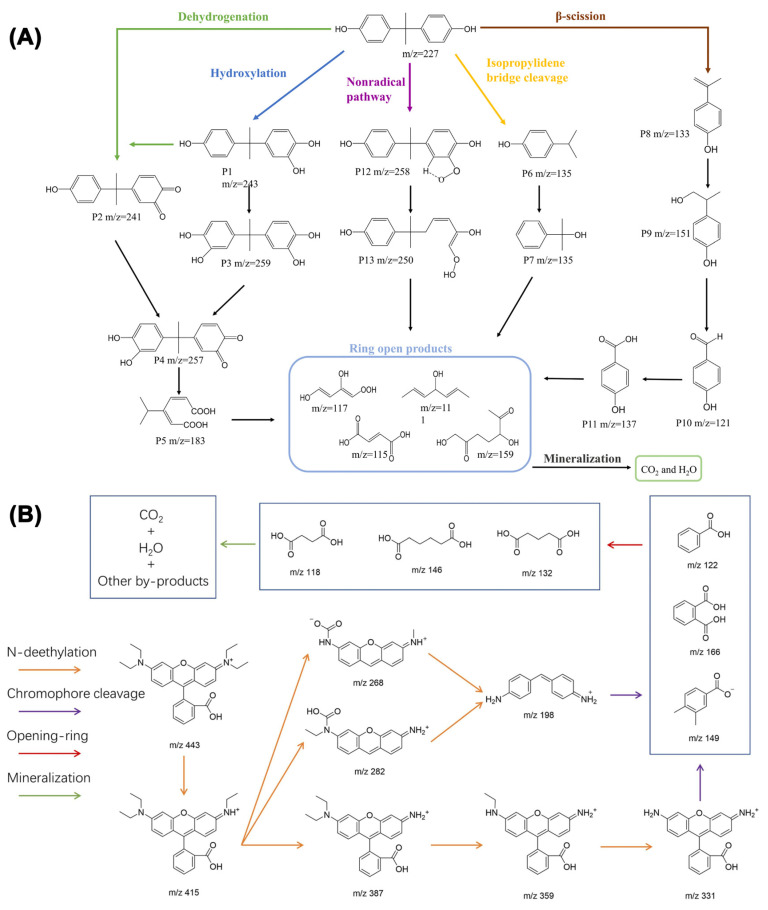
(**A**) Possible degradation pathway of BPA in nZVI@NBC-conducted AOP system [77], Copyright 2022 Elsevier. (**B**) Degradation pathways of the RhB in nZVI/MoS_2_-conducted AOP system [84], Copyright 2023 Elsevier.

The organic contaminants, especially macromolecular compounds, cannot be directly oxidized to CO_2_ and H_2_O with ROS. In the proposed degradation process, these macromolecular compounds firstly suffer from the attack of ROS and form a variety of intermediates, which are subsequently oxidized into small intermediates and gradually decomposed to much smaller intermediates, and are ultimately mineralized to CO_2_, H_2_O, or/and inorganic salts. The suggested degradation mechanisms of RhB and BPA are shown in Figure 7 [77,84].

### 2.3. Concepts for the Future

As the degradation efficiency of contaminants heavily relies on the dissolution of nZVI and the subsequent generation of ROS, a comprehensive study on the formation and transformation mechanisms of ROS in terms of the size of nZVI, surface functionality of nZVI, doping of other components, solution pH, support properties, and molar ratio of nZVI to persulfate may be beneficial for the design and preparation of reasonable nZVI materials for AOPs.

Regarding the practical application in actual water, future research should focus on the precise impact of anions and natural organics on the catalytic activity of nZVI, and the complicated mechanisms involved within. Additionally, the application of nZVI-based AOPs in the synchronous remediation of multiple organic pollutants and the potential novel mechanism involved also deserve to be explored. Moreover, as the recovery of the catalytic activity after repeated use was commonly low in the reported research, nZVI-based catalysts with a higher stability and reusability are required.

## 3. Hydrogen Peroxide-Based AOPs

As an environmentally friendly and cost-effective oxidant, H_2_O_2_ is also widely used as an oxidant in AOPs, since the decomposition products of H_2_O_2_ are harmful H_2_O and O_2_ ions [106]. The traditional Fenton process adopts ferrous salts as catalysts for the activation of H_2_O_2_, which not only requires a limited pH range (2.0–4.0), but also requires a large amount of ferrous salts, leading to the formation of massive sludge and a wastage of iron [107]. Ferrous ions generated from the slow dissolution of nZVI can promise the production of ROS (e.g., •OH, etc.) in the presence of H_2_O_2_, making nZVI a promising and applicable activator in H_2_O_2_-based AOPs [108].

### 3.1. Applications

Until now, many research studies have adopted nZVI in the effective degradation of organic pollutants in H_2_O_2_-based AOPs. The applications of H_2_O_2_-based AOPs conducted with nZVI are summarized in Table 2. Dispersed nZVI or the nZVI supported with porous materials (i.e., rGO, CF, BC, mesoporous hydrated silica (MHS), montmorillonite (MMT)) were adopted for the activation of H_2_O_2_ for the degradation of organic pollutants, such as venlafaxine, citalopram, paroxetine, naproxen, lamotrigine, patulin, ornidazole, acid red 14, polycyclic aromatic hydrocarbons (PAHs), 2,3′,4,5-tetrachlorobiphenyl (PCB67), TBBPA, 1,2-dichloroethane (1,2-DCA), methylene blue (MB), direct red 80, amoxicillin, naphthalene, *p*-nitrophenol (*p*-NP), 2,4-dichlorophenol (2,4-DCP), refractory organic matter, glyphosate, reactive blue 4, and 1,2,3-trichloropropane (TCP) (Table 2).

**Figure 8:**
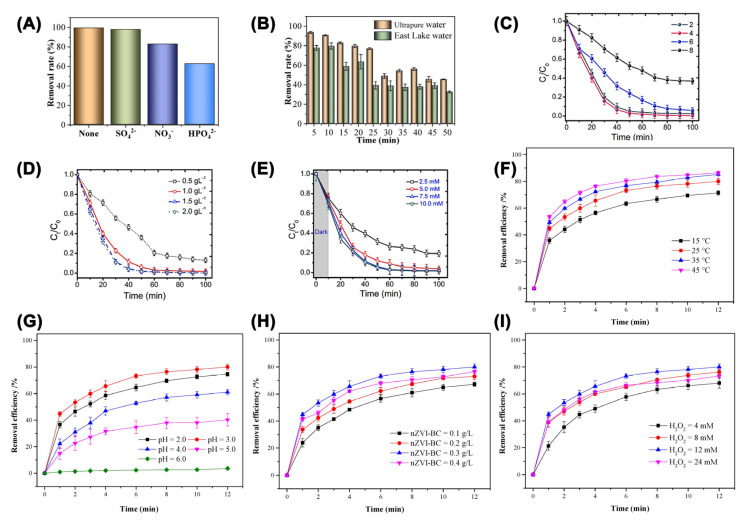
(**A**) Effects of different inorganic anions on the degradation of *p*-NP; (**B**) catalytic performance of rGO/PPy/nZVI catalytic microreactor in ultrapure water and East Lake water for long-term running [120], Copyright 2023 Elsevier. Effects of (**C**) pH, (**D**) catalyst dosage, and (**E**) concentration of H_2_O_2_ on the degradation of MB [116], Copyright 2022 Elsevier. Effects of (**F**) temperature, (**G**) initial pH, (**H**) nZVI-BC dosage, and (**I**) H_2_O_2_ concentration on the degradation of ornidazole [110], Copyright 2020 MDPI.

**Figure 9:**
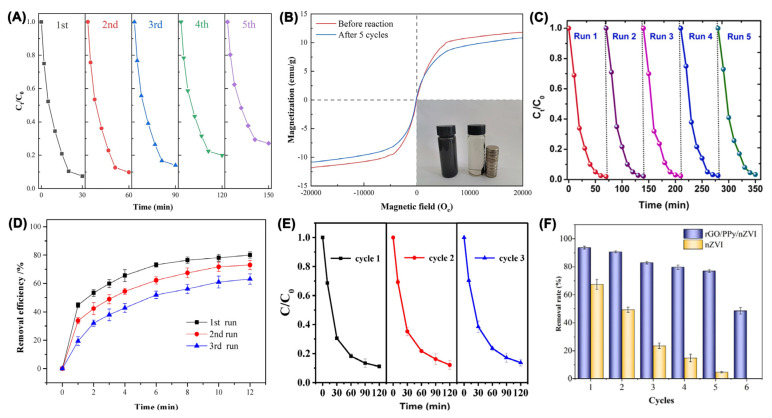
(**A**) Effects of PEG-nZVI@BC recycling on the degradation efficiency of 2,4-DCP; (**B**) the hysteresis loops of fresh and used PEG-nZVI@BC [121], Copyright 2023 Springer. (**C**) Regeneration and reusability of nZVI-FBC nanocomposite [116], Copyright 2022 Elsevier. (**D**) Recycling degradation of ornidazole with nZVI-BC [110], Copyright 2020 MDPI. (**E**) Three cycles of degradation of SMX [129], Copyright 2023 MDPI. (**F**) Long-term stability of rGO/PPy/nZVI and nZVI microreactor [120], Copyright 2023 Elsevier.

In H_2_O_2_-based AOPs conducted with nZVI, a low pH and high temperature are favorable for the degradation of contaminants (Figure 8) [110,116,120]. When the pH is <3.0, excessive H^+^ will act as a •OH scavenger, leading to a decrease in the degradation efficiency [110,116,120]; however, in alkaline conditions, the corrosion of Fe^0^ is limited by an insufficient amount of H^+^ ions, and less •OH are generated from Fe^2+^, in addition to the low oxidation potential of •OH at a high pH and the precipitation of FeOOH on the surface of nZVI, which results in a low degradation efficiency in alkaline conditions [110,116,120]. Similarly, the dosage of nZVI and H_2_O_2_ showed an optimal interval on the degradation efficiency of contaminants, which can be significantly affected by natural organic compounds and inorganic anions as well (Figure 8). A reusability investigation indicated that nZVI materials had preferable recycling performance (Figure 9). Additionally, microwave irradiation, a magnetic field, an electric field, and UV light can be employed to enhance the performance of the Fe^0^/ H_2_O_2_ system [122,126,127].

**Figure 10:**
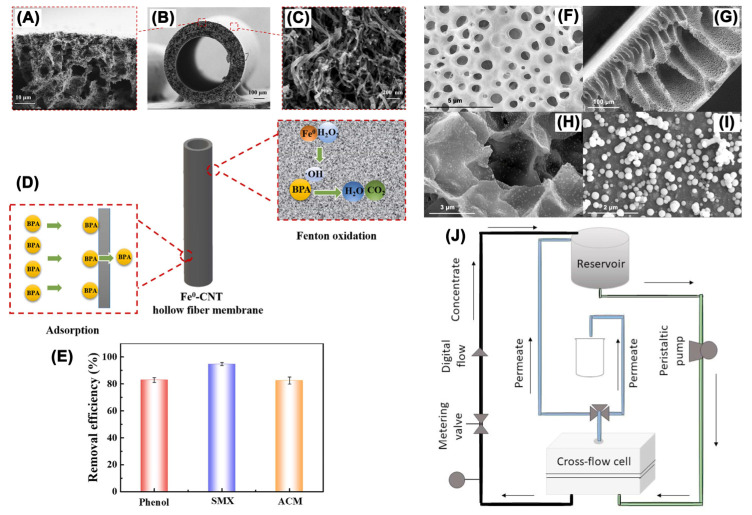
SEM image of Fe^0^-CNTs membrane: cross-section (**A**,**B**), outside surface (**C**); (**D**) mechanism of Fe^0^-CNTs catalytic membrane for BPA wastewater treatment; (**E**) removal of phenol, SMX, and paracetamol (ACM) with Fe^0^-CNTs catalytic membrane system [94], Copyright 2022 Elsevier. SEM images of (**F**) upper surface, (**G**) cross-section, and (**H**,**I**) Fe NPs of nZVI-PVDF_MW_ membrane; (**J**) scheme of cross-flow system employed for BPA removal experiments [128], Copyright 2021 Elsevier.

It is noteworthy to state that nZVI can be immobilized on carbon nanotubes (CNTs) and fabricated as a hollow fiber membrane for the degradation of organic contaminants in flow-through AOPs (Figure 10) [94]. The novel AOP system utilized in this study achieved a high removal efficiency of 98% for BPA, 80.6% for phenol, 95.5% for SMX, and 84.1% for paracetamol under optimal operation conditions (Figure 10E). Silva et al. decorated nZVI on polyacrylic acid which was modified with a hydrophilic polyvinylidene fluoride membrane (nZVI-PVDF_MW_), and this system demonstrated a removal efficiency of 52% ± 0.5 for BPA under a low permeate flux (50 L/(m^2^ h)) in the presence of H_2_O_2_ (Figure 10) [128].

**Figure 11:**
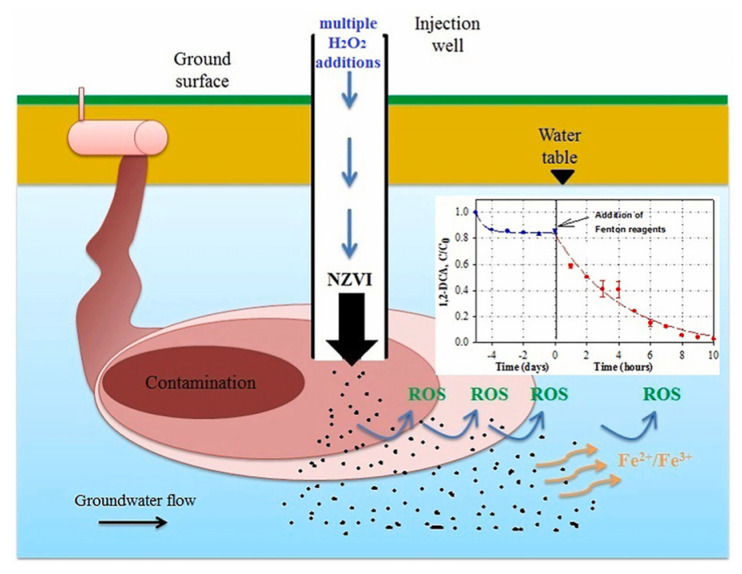
Scheme of the degradation of 1,2-DCA w nZVI/H_2_O_2_ in source zones [115], Copyright 2022 Elsevier.

Le et al. developed a sequential treatment process using an nZVI-induced Fenton-like reaction, overcoming the strict requirements of the traditional Fenton reaction on acidification, and achieved an effective degradation of 1,2-DCA in source zones at an initially neutral pH range (Figure 11) [115]. Chen et al. constructed a novel reaction system with a microreactor and rGO/PPy/nZVI (PPy: polypyrene), which can efficiently catalyze the removal of *p*-NP (>99%, within 50 s), outperforming traditional batch reactors [120].

Besides the removal of contaminants, the combination of nZVI and H_2_O_2_ in AOPs had been adopted to eliminate the harmful cyanobacterium *Microcystis aeruginosa* and the algal organic matters which they produced [106]. This process can effectively destroy the antioxidant enzyme system and then inactivate the cyanobacterial cells, demonstrating their potential application in the removal of *Microcystis aeruginosa* and the algal organic matters.

### 3.2. Mechanisms

nZVI-based AOPs appear to be a viable alternative to the conventional Fenton reaction [115]. In this process, nZVI reacts with H_2_O_2_/H_2_O to release Fe^2+^, providing a source of Fe^2+^ for the generation of •OH, which acts as a primary ROS responsible for the degradation of contaminants (Table 2) (Equations (1), (38) and (39)), while an excessive iron source and H_2_O_2_ may act as a •OH scavenger, thus prohibiting the degradation of contaminants (Equations (40)–(43)) (Figure 8) [110,115,116]. Therefore, a high dosage of H_2_O_2_ or nZVI in AOPs is usually not recommended. A hydroperoxyl radical (•OOH) can also be generated during the activation of H_2_O_2_ with nZVI and the reduction of Fe^3+^ (Equation (42)) [127]. However, compared to •OH (E^0^ = 1.9–2.7 V), •OOH is less reactive and less effective (E^0^ = 1.44 V) [130,131].

Additionally, employing external energy, especially UV radiation, a magnetic field, and microwave irradiation, can accelerate the corrosion of nZVI and enhance the activation of H_2_O_2_ (Equations (44) and (45)) [111,117,122,126].

Fe^0^ + H_2_O_2_ + 2H^+^ → Fe^2+^ + 2H_2_O (38)

Fe^2+^ + H_2_O_2_ → Fe^3+^ + OH^−^ + •OH (39)

•OH + Fe^2+^ → OH^−^ + Fe^3+^ (40)

•OH + •OH → H_2_O_2_ (41)

•OH + H_2_O_2_ → H_2_O + •OOH Fe^3+^ + H_2_O_2_ → Fe^2+^ + •OOH + H^+^ (42)

•OOH + •OH → H_2_O + O_2_ (43)

H_2_O_2_ + *hv* → 2•OH (44)

Fe(OH)^2+^ + *hv* → Fe^2+^ + •OH (45)

### 3.3. Concepts for the Future

As the concentrations of H_2_O_2_ in nZVI-based AOP systems were usually overdosed to obtain a satisfied degradation efficiency, future research should focus on the optimization of the pH level, reactivity of nZVI (size, modification, or doping), dosage strategy of nZVI, and other reaction conditions, committing to advancement of the removal efficiency in a moderate nZVI/H_2_O_2_ dosage, and improving the recyclability of the nZVI materials. Furthermore, the combination of nZVI and H_2_O_2_-based AOPs for wastewater remediation in novel reaction systems, such as membrane filtration, cross-flow system, and microreactor, also deserves to be continuously and thoroughly investigated.

## 4. Oxygen-Based AOPs

Molecular oxygen can be activated with enzymes in vivo and in vitro for the oxidation of the relevant organic functional metabolites. Moreover, it is difficult to force oxygen to directly react with most organic contaminants in ambient conditions. This property of oxygen is caused by its diradical stable structure with a triplet ground state [132]. Since Noradoun et al. discovered that molecular oxygen in an aerobic solution can be activated with Fe^0^ and applied in the degradation of organic pollutants under ambient conditions, many research studies have been conducted on the oxygen activation process with the use of Fe^0^ and hence promoted the development of oxygen-based AOPs conducted with nZVI for environmental remediation [133]. Therefore, studies adopting nZVI on the in situ generation of H_2_O_2_ and ^1^O_2_ in AOPs have received more and more attention in recent years.

### 4.1. Applications

As shown in Table 3, O_2_-based AOPs conducted with nZVI were employed in the degradation of ciprofloxacin, OTC, chloramphenicol, MB, brilliant green, nitrobenzene, atrazine, SMX, etc. To overcome the disadvantages of the agglomeration and oxidation of nZVI, efforts of coating, supporting, doping, and encapsulating were applied to obtain high reactivity and high stability nZVI materials for O_2_-based AOPs. Yang et al. constructed a ternary nanocomposite of 3D-rGO@nZVI/Al_2_O_3_, achieving an efficient removal of chloramphenicol with the motivation of dissolved oxygen (DO) [134]. As the concentration of contaminants in wastewater may be low for Fenton degradation, researchers developed nZVI materials with a high adsorption capability, aimed at enriching the contaminants on a catalyst to enable subsequent degradation [135,136,137]. Cheng et al. adopted an amorphous nZVI material as the catalyst of O_2_-based AOPs for the oxidation of Sb(III), and the aged amorphous nZVI can still work as an adsorbent for the removal of Sb(IV) [138].

**Figure 12:**
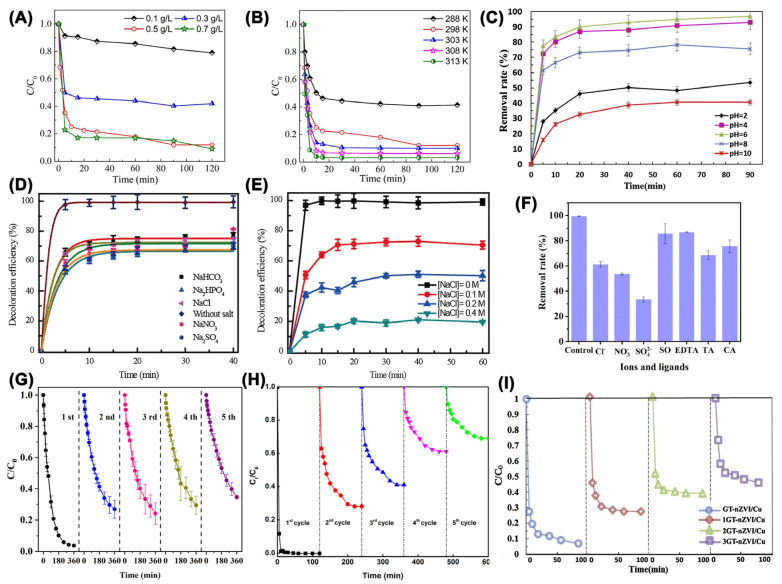
(**A**) Effects of dosage of 3D-GN@Fe/Al and (**B**) temperature on chloramphenicol removal [132], Copyright 2021 Elsevier. (**C**) Effects of initial pH on catalytic reactivity of GT-nZVI/Cu [139], Copyright 2020 Elsevier. (**D**) Effects of anions on MB decolorization efficiency ([salt]  =  0.1 M) and (**E**) effects of ionic strength on MB decolorization efficiency [137], Copyright 2021 Springer. (**F**) Influences of co-existing ions and organic ligands on ciprofloxacin removal; (**G**) recycling of nZVI/PA composites [107], Copyright 2023 MDPI. (**H**) Reusability of the Pt/nZVI composite [140], Copyright 2020 Elsevier. (**I**) Effects of regeneration times on catalytic reactivity [139], Copyright 2020 Elsevier.

Concerning the influence of reaction factors on the removal of contaminants, a high temperature and a moderately acid condition is favorable (Figure 12) [132,139]. When the pH is too low, the generated H_2_ bubbles may cover the surface of nZVI, preventing the working ability of nZVI-based AOPs and further decreasing the removal efficiency of contaminants [139]. Furthermore, a basic solution is not only adverse to the corrosion of nZVI, but also unfavorable for the adsorption of negatively charged contaminants [140]. The removal efficiency of contaminants can also be significantly affected by inorganic anions and organic compounds (Figure 12) [107,137,138]. Furthermore, the reusability investigation indicated that the removal efficiency decreased significantly in the recycling process of nZVI materials, which may be attributed to the formation of iron oxide on nZVI, resulting from the deactivation of nZVI with DO (Figure 12) [107,139,140].

### 4.2. Mechanisms

**Figure 13:**
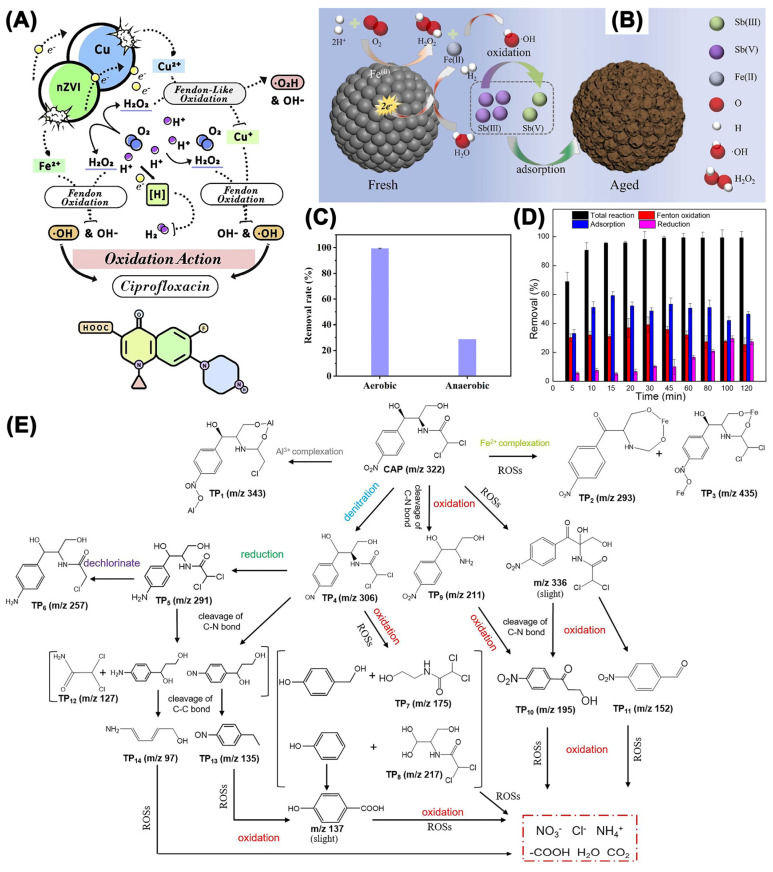
(**A**) Schematic model for removing ciprofloxacin by GT-nZVI/Cu under aerobic conditions [139], Copyright 2020 Elsevier. (**B**) Reaction mechanism of A-nZVI and Sb(III) [138], Copyright 2022 Elsevier. (**C**) Effects of DO on ciprofloxacin removal [107], Copyright 2022 MDPI. (**D**) Contributions of adsorption, Fenton reaction, and reduction to overall OTC removal using Pt/nZVI composite [140], Copyright 2020 Elsevier. (**E**) Possible degradation pathway of chloramphenicol with 3D-GN@Fe/Al [132], Copyright 2021 MDPI.

In O_2_-based AOPs, it is worth noting that nZVI can react with DO through a two-electron reduction reaction to generate H_2_O_2_ (Equation (46)), and can further generate •OH via the Fenton reaction (Equation (47)) [141,142]. The equations and reported studies indicated that the generation of •OH prefers an acid condition [107,143], and DO is essential in the formation of •OH, and was the primary factor responsible for the oxidation of contaminants and their intermediates (Figure 13) [143]. With regard to the degradation mechanism of contaminants, nZVI can also exert adsorption, reduction, dichlorination, and denitration on contaminants, promoting the mineralization process of contaminants (Figure 13B,D) [132,141].

The reaction of Fe^2+^ and O_2_ may produce •O_2_^−^, which can be further ignited to form the more reactive ^1^O_2_ ions (Equations (48) and (49)) [134,144]. Many researchers have unveiled the important role of •O_2_^−^ and ^1^O_2_ in the oxidative degradation of contaminants and their generation routes that are assisted by doped Al/Mn (Equations (50)–(52)) [107,132,134,142,143].

2Fe^0^ + O_2_ + 2H^+^ → Fe^2+^ + H_2_O_2_ (46)

Fe^2+^ + H_2_O_2_ → Fe^3+^ + OH^−^ + •OH (47)

Fe^2+^ + O_2_ → Fe^3+^ + •O_2_^−^ (48)

2H^+^ + 2•O_2_^−^ → ^1^O_2_ + H_2_O_2_ (49)

Mn^3+^ + H_2_O_2_ → Mn^4+^ + OH^−^ + •OH (50)

Al^3+^ + •O_2_^−^ → •AlO_2_^2+^ (51)

Fe^3+^ + •AlO_2_^2+^ → Fe^2+^ + Al^3+^ + ^1^O_2_ (52)

### 4.3. Concepts for the Future

DO plays a key role in the corrosion of nZVI and the production of ROS; however, few studies have applied it in the exploration of the influence of factors relating to DO (i.e., concentration) on the generation of ROS and the removal efficiency of contaminants.

nZVI materials are prone to being oxidized by a dissolved oxygen ion and form iron oxides on the surface of nZVI, thus suppressing the reactivity of nZVI and diminishing the remediation performance. This requires a systematical investigation of the comprehensive effects of pH, DO, temperature, reactivity of nZVI, and the physicochemical characteristic of contaminants on the remediation efficiency, which may be beneficial for achieving optimal remediation conditions in practical applications.

Furthermore, as a residue-free approach for wastewater remediation, O_2_-based AOPs deserve to be more intensively studied in theoretical and practical aspects.

## 5. Other Oxidant-Based AOPs

### 5.1. Peracetic Acid-Based AOPs

Peracetic acid (PAA) is extensively used as a disinfector in hospital, tableware disinfection, food processing, and sewage processing due to its highly efficient and broad-spectrum bactericidal capacity and low possibility to form mutagenic and carcinogenic disinfection byproducts during application [145]. PAA has high oxidation–reduction potential (1.96 V) comparable to H_2_O_2_ (1.78 V) and persulfate (2.01 V), and a lower bond energy of O−O than H_2_O_2_ and persulfate, making it a promising oxidant for AOPs [146].

Wang et al. adopted nZVI to activate PAA under ultraviolet (UV) light for the degradation of spiramycin (10 mg/L), which achieved a complete removal of spiramycin in 20 min [147]. Results of quenching experiments and EPR analysis confirmed that 52.4% and 44.8% of spiramycin removal was attributed to •OH and carbon-centered radicals (•C−H) (Equations (53)–(56)), respectively. And, the Fe^2+^ released from nZVI played a critical role in the generation of these radicals.

Fe^2+^ + CH_3_COOOH → Fe^3+^ + CH_3_COO ^−^ + •OH (53)

Fe^2+^ + CH_3_COOOH → Fe^3+^ + •CH_3_COO + OH^−^ (54)

•CH_3_COO → •CH_3_ + CO_2_ (55)

•OH + CH_3_COOOH → •CH_3_CO + O_2_ + H_2_O (56)

•OH + CH_3_COOOH → •CH_3_COOO + H_2_O (57)

Co^2+^ + CH_3_COOOH → Co^3+^ + •CH_3_COO + OH^−^ (58)

Co^3+^ + CH_3_COOOH → Co^2+^ + •CH_3_COO + H^+^ (59)

Fe^3+^ + CH_3_COOOH → Fe^2+^ + •CH_3_COO + H^+^ (60)

**Figure 14:**
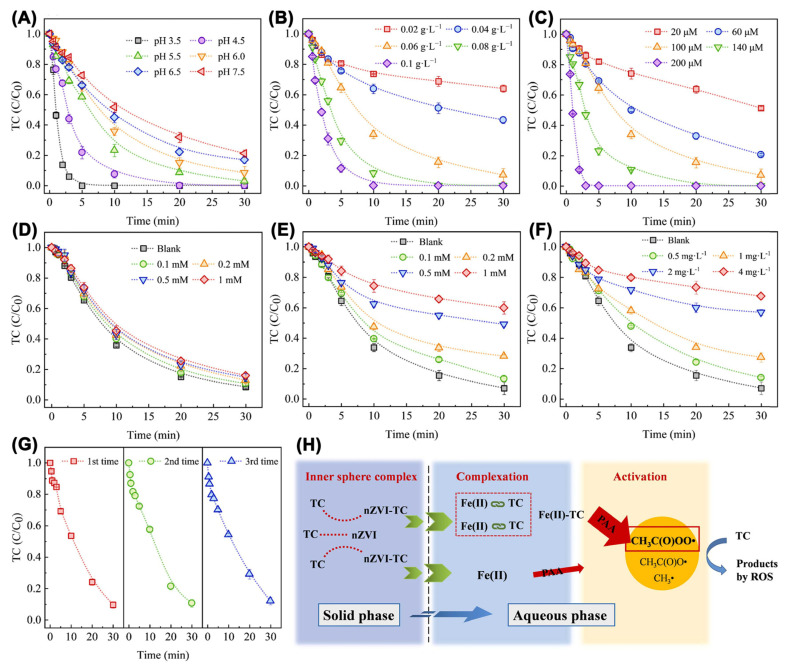
Effects of (**A**) solution pH, (**B**) nZVI dosage, and (**C**) PAA concentration on TC abatement in nZVI/PAA process; effects of (**D**) Cl^−^, (**E**) HPO_4_^2−^, and (**F**) HA on TC abatement; (**G**) performance of reusable nZVI on TC abatement; (**H**) proposed mechanism for activation of PAA with Fe(II)-TC complexes in nZVI/PAA process [148], Copyright 2022 Elsevier.

Zhang et al. constructed an AOP with PAA and nZVI, achieving TC abatement efficiencies with pH levels of pH 3.5 (100%) = pH 4.5 (100%) > pH 5.5 (96.6%) > pH 6 (92.9%) > pH 6.5 (86.7%) > pH 7.5 (79.4%) under 25 °C after 30 min of reaction, in this order (Figure 14) [148]. The degradation of TC prefers a high concentration of PAA and nZVI, while the gradation can be inhibited with inorganic ions and HA in varying degrees (Figure 14). The most dominant radical responsible for the TC abatement was identified as •O_3_CCH_3_ (Equation (57)), which mainly originates from the homogeneous activation of PAA with Fe(II) and Fe(II)-TC complexes. Fe(II)-TC complexes with ligands containing N– and O– functional groups play an important role in the homogeneous and heterogeneous PAA activation processes (Figure 14). Moreover, the degradation efficiency of TC remained at ~90% after being recycled three times, indicating that nZVI has good reusability for PAA activation in AOPs.

Another research study conducted by Yang et al. discovered that Co-doped Fe^0^ can generate multiple types of ROS during the activation of PAA, including organic radicals (•O_3_CCH_3_, •O_2_CCH_3_, •CH_3_) and •OH [146]. Fe(III)/Fe(II) and Co(III)/Co(II) redox cycles can facilitate the formation of ROS (Equations (54) and (58)–(60)). The degradation of SMX was dominated by organic radicals in the first period (0–10 min), while •OH took the main role in SMX degradation in the following period (10–30 min).

### 5.2. Periodate-Based AOPs

As a solid-form oxidant, periodate (PI) is stable during storage and transportation, and is regarded as a promising alternative to liquid-form oxidants [149]. The activation of PI can produce a variety of ROS, including a iodate radical (•IO_3_), •OH, atomic oxygen radical anion (•O^−^), ^1^O_2_, and •O_2_^−^ [149]. Zong et al. employed nZVI for the activation of PI, which achieved an effective oxidization of sulfadiazine [149]. This nZVI/PI process obtained excellent resistance to the interference of coexisting substances (Cl^−^, CO_3_^2−^, HA) and to pH variations (4.0–7.0). The production of •IO_3_ and •OH (Equations (61) and (62)) followed a surfaced-mediated activation pathway due to the relatively weaker steric hindrance effect of PI, and thus resulted in a relatively long reaction time being required (1–6 h under the tested conditions) for achieving a satisfactory removal efficiency of the target contaminant. Therefore, efforts on the modulation effect of the reactivity and selectivity of nZVI are still required to enhance the oxidization capacity of the nZVI/PI process.

Fe^2+^ + IO_4_^−^ + 2H^+^ → Fe^3+^ + •IO_3_ + H_2_O (61)

•IO_3_ + H_2_O → IO_3_^−^ + •OH + H^+^ (62)

He et al. prepared Cu-doped nZVI supported by sludge biochar (Fe/Cu-SBC) for the activation of PI to degrade diclofenac sodium with the assistance of UV light at room temperature [150]. A total of 99.7% of diclofenac sodium was degraded in 60 min under an optimized condition. Radical scavenging and gas purging experiments indicated that •IO_3_ radicals were predominantly responsible for the oxidation of diclofenac sodium.

### 5.3. Percarbonate-Based AOPs

Percarbonate, known as solid H_2_O_2_, has emerged as a desirable oxidant in AOPs due to its advantages of being more stable, having a wider pH range of applicability, being lower cost, nontoxic, and environmentally friendly [151,152]. Additionally, the byproducts in the decomposition of percarbonate are low-hazardous Na_2_CO_3_ and H_2_O_2_; thus, percarbonate was suggested as a novel source of H_2_O_2_ (Equation (63)).

2Na_2_CO_3_**·**3H_2_O_2_ → 2Na_2_CO_3_ + 3H_2_O_2_ (63)

•OH + CO_3_^2−^ → OH^−^ + •CO_3_^−^ (64)

H_2_O_2_ + •CO_3_^−^ → HCO_3_^−^ + •OOH (65)

•OOH → H^+^ + •O_2_^−^ (66)

**Figure 15:**
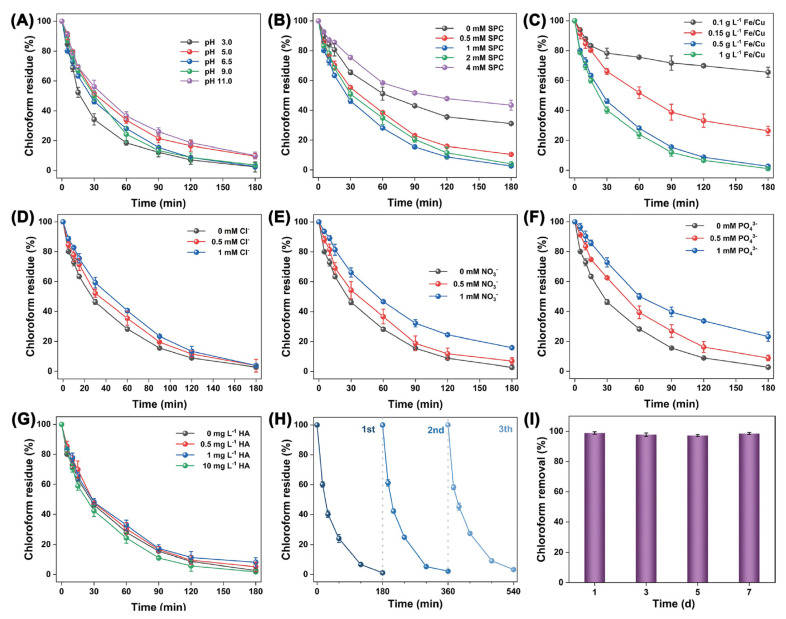
Effects of (**A**) initial pH, (**B**) sodium percarbonate dosage, (**C**) TOCNF-Fe/Cu dosage, (**D**) Cl^−^ concentration, (**E**) NO_3_^−^ concentration, (**F**) PO_4_^3−^ concentration, and (**G**) HA concentration on chloroform removal; (**H**) recyclability and (**I**) stability of TOCNF-Fe/Cu [151], Copyright 2023 Elsevier.

Che et al. prepared cellulose nanofiber-supported iron/copper bimetallic NPs (TOCNF-Fe/Cu) to activate sodium percarbonate for the removal of chloroform from groundwater [151]. The system resulted in a removal efficiency of >97.3% of CF in a neutral reaction medium (pH 6.5–9) within 180 min (Figure 15) with •O_2_^−^ and •OH (Equations (64)–(66)), the former of which was identified as the primary ROS for the degradation of CF. The degradation of chloroform with TOCNF-Fe/Cu favors a moderate concentration of nZVI and sodium percarbonate, rather than an excessive dosage (Figure 15). The result also indicated that inorganic ions and HA can impose unfavorable effects on the degradation efficiency (Figure 15). Interestingly, this nZVI-based catalyst performed well in terms of recyclability and stability, and retained its activity after three cycles and even one week of aging (Figure 15).

Rashid et al. adopted natural zeolite as a support to load nZVI (NZ-nZVI) to activate sodium percarbonate for the removal of MO, and more than 90% of dyes can be removed within 180 min using this approach [153]. Xiao et al. investigated multiple influencing factors (nZVI loading, sodium percarbonate dosing, initial pH, the presence of inorganic anions, and humic acid) on BPA removal with the combination of nZVI and sodium percarbonate [152]. This work indicated that the degradation efficiency of BPA can be enhanced with increased nZVI loading and sodium percarbonate dosing in the adequate range at a low initial pH; the presence of inorganic anions (Cl^−^, HPO_4_^2−^, NO_2_^−^) and HA in an aqueous solution inhibited the removal of BPA in the selected range. Moreover, the activation of sodium percarbonate, which is attributed to (i) the surface corrosion of nZVI, (ii) Fe^2+^ release, and (iii) conversion of iron species, can generate •OH and •CO_3_^−^, the former of which was the dominant active species, while the latter contributed only slightly to the degradation of BPA.

### 5.4. Concepts for the Future

As actual polluted water or soil always contains various organic contaminants, future research should focus on the applications of nZVI in complex pollution scenarios via AOPs, adsorption-AOPs, adsorption-enrichment-AOPs, or other effective pathways.

Compared to the simple use of a single oxidant, some research studies that adopted two or more oxidants in AOPs showed an excellent degradation efficiency in contaminants [154,155,156]. Thus, more exploration is needed to gain an in-depth understanding of the synergistic effects among various oxidants, degradation mechanisms, and their feasibility. Additionally, a comprehensive understanding of reaction kinetics, intermediate products, and mechanisms of AOPs conducted with nZVI and multiple oxidants should be clarified to provide guidance for the designing of nZVI materials with a high activation efficiency on specific oxidants.

## 6. Conclusions and Prospectives

The extensive development and utilization of organic products give rise to more and more antibiotics, pharmaceuticals, dyes, pesticides, PAHs, organic chlorides, phenols, and so on being transported into water and soil, forming environmental contaminants. Rapid and complete removal of these organic contaminants appears to be significant and urgent due to them providing a toxicity risk to the environment and to human beings. The combination of nZVI and AOPs for the removal of these organic pollutants is attracting increasing attention. In order to obtain a higher removal efficiency and a better degradation performance on the contaminants, many studies have been conducted on the AOPs that can be catalyzed with nZVI materials. However, synthesizing mono-disperse nZVI, retaining their surface activity, and avoiding aggregation during AOPs is still challenging, and is not only attributed to the strong van der Waal and magnetic attraction forces of nZVI, but also due to nZVI being prone oxidization via water, even in an anoxic condition. Therefore, doped, coated, and supported nZVI materials have been synthesized to overcome these shortages, and to obtain a better catalytic activity and reusability.

These modified nZVI with enhanced dispersibility and reusability have been applicated in multiple oxidants that conducted AOPs, such as persulfate, H_2_O_2_, O_2_, peracetic acid, periodate, and percarbonate, for the degradation of various organic contaminants. The mechanisms involved in nZVI-based AOPs are discussed. The degradation of these organic contaminants mainly depends on the indiscriminate oxidation of organics with ROS, such as •OH, •SO_4_^−^, •O_2_^−^, •O_3_CCH_3_, •O_2_CCH_3_, •IO_3_, •CO_3_^−^, and ^1^O_2_, which are generated from Fenton-type reactions. Benefiting from their extremely high oxidization ability, the pollutants can be ultimately mineralized into inorganic products, including CO_2_, H_2_O, or salts.

However, there are still some challenges in the employment of nZVI materials in AOPs to degrade contaminants:

(1) The applications of nZVI-based materials in AOPs for the removal of organic pollutants still lack accurate models and reaction kinetics regarding the dissolution and catalyzation processes of nZVI. Quantitatively exploring and establishing these models and kinetics will provide theoretical guidance on the fabrication of more effective and sustainable nZVI materials, optimization of the reaction conditions, or a more reasonable dosage strategy of nZVI during AOPs.
(2) A few research studies reported the combination of multiple oxidants in nZVI-based AOPs, in which the reaction mechanism involved is complicated and less understood. Therefore, employing multiple oxidants in AOPs conducted with nZVI calls for more studies to verify the potential enhancement effects on the oxidization ability toward contaminants, and would enable us to obtain in-depth knowledge of the interactions among various oxidants, uncharted degradation mechanisms, and their feasibility.
(3) In nZVI-based AOPs, organic pollutants can be theoretically degraded and mineralized into harmful CO_2_ and H_2_O, although many reported research studies were mainly focused on the removal efficiency of the target pollutants. Future studies may need to stress the complete degradation of the pollutants, such as committing to reduction of the total organic carbon (TOC) value of the polluted water during AOPs, and clarifying the potential toxic aspects of intermediate products via toxicity studies.
(4) Although this research results confirmed the feasibility of nZVI-based AOPs for the degradation of contaminants, there is a large gap between laboratory level research and the remediations aimed towards actual polluted water. In addition to actual polluted water containing a variety of pollutants, coexisting inorganic ions, and interfering matter, present works were usually carried out on simulated wastewater, and thus research studies that are dedicated to the applicability of nZVI-based AOPs in actual situations are necessary.
(5) There is a need for further studies to obtain a greater understanding on the potential synergistic and antagonistic effects among contaminants, as well as their intermediate products during their oxidative degradation with ROS.
(6) The recoverability and reusability of nZVI materials is an important characteristic that is crucial to the practical application on a large scale, since an appropriate catalyst that is applicable in AOPs under real conditions is required to be reasonable, durable, and convenient in operation. The recovery of the catalytic activity of nZVI after repeated use was commonly low in the existing research. Therefore, further exploration into nZVI materials with a controllable discharge of Fe^2+^, prolonged stable catalytic activity, favorable separation, and excellent recyclability is fascinating.
(7) Extending the application of nZVI-based AOPs in high-value fields, such as preventing antimicrobial resistance emergence and biofilm formation [157], or biomedical applications, is also worthy of being studied.
(8) Finally, studies on the techno-economic and environmental impact of nZVI-based AOPs by means of life cycle analysis are also worthwhile to conduct before undertaking practical applications.

## Figures and Tables

**Table 1:** Literature studies on persulfate-based AOPs from the last three years. The operation conditions comprise reaction temperature (°C), mole ratio of nZVI to PS, and pH, respectively. The symbol “--” represents the related data that were not mentioned in the reference.

Catalyst	Oxidant (Concentration, mM)		Pollutant (Concentration, mM)		Operation Conditions (°C/Mole Ratio/pH)			Dominant Radicals	Removal Efficiency	Catalyst Reusability	Ref.
BC-nZVI	--	--	Petroleum	--	25	1:1	--	•SO_4_^−^	50% (40 h)	--	[56]
Fe^0^@C	Na_2_S_2_O_8_	1.0	4-CP	0.15	25	1.75:1	5.7	•OH > •SO_4_^−^	82% (15 min)	--	[57]
nZVI-rGO	Na_2_S_2_O_8_	6.5	Nonylphenol	0.09	30	1.26:1	4.2	•SO_4_^−^	98.2% (50 min)	5	[58]
nZVI-rGO	Na_2_S_2_O_8_	4.28	Furfural	2.6	70	3.97:1	5.5	•SO_4_^−^ > •OH	97.8% (50 min)	5	[59]
nZVI/Co_3_O_4_	K_2_S_2_O_8_	0.90	TC	0.045	25	3.98:1	3.0	•SO_4_^−^	97.6% (15 min)	4	[60]
nZVI	--	2.0	TPHP	0.006	30	1:4	3.0	•SO_4_^−^	98.2% (60 min)	--	[61]
BC@nZVI/Ni	Na_2_S_2_O_8_	0.6	Norfloxacin	0.031	30	0.82:1	3.0	•SO_4_^−^	99% (30 min)	--	[62]
AC-nZVI	--	1.0	Ampicillin	0.05	60	0.25:1	--	•OH > •SO_4_^−^	~99% (60 min)	5	[63]
nZVI	Na_2_S_2_O_8_	1.0	SMZ	0.19	25	2:1	6.8	•SO_4_^−^ and •OH	96% (30 min)	--	[64]
nZVI	Na_2_S_2_O_8_	1.0	SMX	0.0395	25	1.79:1	5.53	•SO_4_^−^ and •OH	88.4% (2 h)	--	[65]
nZVI	Na_2_S_2_O_8_	1.9	Petroleum	--	25	0.28:1	4.2	•SO_4_^−^ > •OH	61.2% (2 h)	5	[66]
TiO_2_@nZVI	K_2_S_2_O_8_	0.5	Amoxicillin	0.055	--	--	5.0	•SO_4_^−^ and •OH	99% (60 min)	4	[67]
nZVI@CS	--	1.0	Atrazine	0.0464	25	1.125:1	7.0	•SO_4_^−^ > •OH	96.65% (60 min)	4	[68]
nZVI/CF	KHSO_5_	1.0	Levofloxacin	0.05	25	2.86:1	7.0	•SO_4_^−^ > •OH	93.83% (60 min)	--	[69]
nZVI@BC	Na_2_S_2_O_8_	1.0	Atrazine	0.046	25	0.2:1	5.0	•SO_4_^−^	93.8% (30 min)	4	[70]
PVP-nZVI-Cu	Na_2_S_2_O_8_	6.0	TCE	0.15	25	0.25:1	3.2	•SO_4_^−^ and •OH	99.6% (60 min)	4	[71]
nZVI-Ni@BC	Na_2_S_2_O_8_	4.0	TCE	0.15	--	0.18:1	~3.49	•SO_4_^−^ and •OH	~99.0% (60 min)	4	[72]
nZVI/CuO@BC	K_2_S_2_O_8_	0.2	TBBPA	0.0184	25	2.2:1	8.0	•SO_4_^−^ and •OH	98.46% (45 min)	--	[73]
CS-PHB-nZVI	K_2_S_2_O_8_	2.0	MO	0.15	25	0.29:1	7.0	•SO_4_^−^ and •OH	~100% (30 min)	4	[74]
C-nZVI	Na_2_S_2_O_8_	1.0	SMZ	0.072	20	1:1	8.3	•SO_4_^−^ and •OH	45.3% (120min)	--	[75]
PDA/ATP-nZVI	Na_2_S_2_O_8_	2.0	8-HQ	0.172	25	2.2:1	3.0	•SO_4_^−^ and •OH	96.6% (35min)	--	[76]
nZVI@NBC	Na_2_S_2_O_8_	1.0	BPA	0.1	25	0.55:1	7.0	SO_4_^−^, •OH, ^1^O_2_	95% (120 min)	3	[77]
nZVI/Mn	--	1.0	SMZ	0.018	30	1.25:1	3.0	•OH	95% (60 min)	--	[78]
nZVI@gBC	Na_2_S_2_O_8_	2.0	2,4-DCP	0.12	26	0.53:1	6.54	•SO_4_^−^	~100% (20 min)	3	[79]
rGOA-nZVI	--	4.0	OPPs	0.038	25	--	5.0	•SO_4_^−^ > •OH	99.5% (5 min)	--	[80]
Fe@MC	Na_2_S_2_O_8_	1.0	TBBPA	0.0184	--	1.79:1	7.0	•SO_4_^−^ and •OH	94.9% (30 min)	4	[15]
MoS_2_/nZVI	K_2_S_2_O_8_	1.2	SMX	0.02	--	1.49:1	4.0	•SO_4_^−^ and •OH	98.6% (30 min)	5	[81]
nZVI-BC	Na_2_S_2_O_8_	6.0	Pyrene	0.049	25	1.79:1	3.0	•SO_4_^−^ and •OH	99.4% (60 min)	--	[82]
nZVI	KHSO_5_	0.9	TCE	0.15	20	2.38:1	5.0	•SO_4_^−^ > •OH	97.8% (15 min)	--	[83]
nZVI/MoS_2_-BC	KHSO_5_	0.325	RhB	0.021	--	--	6.0	•SO_4_^−^ > •OH	90.88% (60 min)	5	[84]
nZVI@P-BC	Na_2_S_2_O_8_	4.0	γ-HCH	0.0344	25	2.8:1	--	•OH and ^1^O_2_	92.6% (10 min)	5	[85]
nZVI-BC	Na_2_S_2_O_8_	0.5	OTC	1.3	25	24:1	--	•SO_4_^−^, •OH, ^1^O_2_	98.34% (5 h)	--	[86]
nZVI-BC	--	3.0	Phenol	0.53	25	0.95:1	7.0	•O_2_^−^ and ^1^O_2_	100% (60 min)	3	[87]
GC-nZVI	KHSO_5_	0.25	BPA	0.025	25	0.25:1	7.0	•SO_4_^−^ > •OH	100% (60 min)	3	[88]
nZVI-Cu^0^	KHSO_5_	0.025	Atrazine	0.0021	25	36:1	4.8	•SO_4_^−^ > •OH	100% (10 min)	5	[40]

**Table 2:** Literature studies on H_2_O_2_-based AOPs from the last three years. The operation conditions comprise reaction temperature (°C), mole ratio of nZVI to H_2_O_2_, and pH, respectively. The symbol “--” represents the related data that were not mentioned in the reference.

Catalyst	Oxidant (Concentration, mM)		Pollutant (Concentration, mM)		Operation Conditions (°C/Mole Ratio/pH)			Dominant Radicals	Removal Efficiency	Catalyst Reusability	Ref.
rGO–nZVI	H_2_O_2_	10	Venlafaxine	0.0007	--	0.73:1	3.0	•OH	93.2% (30 min)	--	[108]
			Citalopram	0.0006					98.51% (30 min)		
			Paroxetine	0.0005					99.34% (30 min)		
			Naproxen	0.0009					99.56% (30 min)		
			Lamotrigine	0.0008					97.9% (30 min)		
CE-nZVI	H_2_O_2_	0.5	Patulin	0.0026	--	1.79:1	3.5	•OH	99.1% (1 min)	--	[109]
							4.5		98.0% (240 min)		
							5.0		87% (240 min)		
							6.0		27.8% (240 min)		
nZVI-BC	H_2_O_2_	12	Ornidazole	0.455	25	0.15:1	3.0	•OH	80.1% (12 min)	3	[110]
nZVI	H_2_O_2_	10	Acid red 14	0.199	22	0.09:1	3.0	•OH	89.3% (90 min)	--	[111]
nZVI	H_2_O_2_	47	PAHs	--	25	1.65:1	2.9	•OH	89.3% (200 min)	--	[112]
MMT-nZVI	H_2_O_2_	676	PCB67	0.0028	28	--	3.5	•OH	76.38% (80 min)	--	[113]
nZVI/Co@mHS	H_2_O_2_	20	TBBPA	0.0184	30	0.11:1	5.0	•OH	97.13% (6 h)	6	[114]
nZVI	H_2_O_2_	25	1,2-DCA	20.21	--	7.2:1	7.0	•OH	99% (16 h)	3	[115]
nZVI-FBC	H_2_O_2_	7	MB	0.0625	25	0.04:1	4.5	•OH	99% (40 min)	5	[116]
EG-nZVI	H_2_O_2_	1.5	Direct red 80	0.0728	25	0.01:1	5.5	•OH	90% (180 min)	--	[117]
		3.5				0.05:1	5.5		~95% (180 min)		
nZVI	H_2_O_2_	888	Amoxicillin	0.274	--	0.01:1	3.0	•OH	99.7% (120 min)	--	[118]
Fe(II)/nZVI	H_2_O_2_	1.0	Naphthalene	0.1	20	0.5:1	5.52	•OH	99% (120 min)	--	[119]
rGO/PPy/nZVI	H_2_O_2_	6.52	*p*-NP	0.072	--	--	3.0	•OH	99.6% (50 s)	--	[120]
PEG-nZVI@BC	H_2_O_2_	4.0	2,4-DCP	0.307	25	2.4:1	3.0	•OH, •O_2_^−^, ^1^O_2_	92.94% (30 min)	5	[121]
nZVI	H_2_O_2_	30	Refractory organic matter	--	--	0.3:1	3.0	•OH	85.79% (60 min)	--	[122]
nZVI	H_2_O_2_	0.3	Glyphosate	0.012	20	0.6:1	3.0	•OH	~100% (30 min)	--	[123]
nZVI-BC	H_2_O_2_	10	Reactive blue 4	0.0785	20	0.09:1	3.2	•OH	99.56% (30 min)	--	[124]
Fe(II)/nZVI	H_2_O_2_	3.0	TCP	0.15	20	0.75:1	5.71	•OH	95.4% (120 min)	--	[125]

**Table 3:** Literature studies on O_2_-based AOPs from the last three years. The operation conditions comprise reaction temperature (°C), concentration of nZVI (C_Fe_, mM), and pH, respectively. The symbol “--” represents the related data that were not mentioned in the reference.

Catalyst	Oxidant	Pollutant (Concentration, mM)		Operation Conditions (°C/C_Fe_/pH)			Dominant Radicals	Removal Efficiency	Catalyst Reusability	Ref.
GT-nZVI/Cu	DO	Ciprofloxacin	0.151	20	8.3	6.0	•OH	86.9% (90 min)	4	[139]
				30				92.9% (90 min)		
				40				~100% (90 min)		
Pt/nZVI	DO	OTC	0.217	25	8.9	5.0	•OH	~100% (20 min)	5	[140]
3D-GN@Fe/Al	DO	Chloramphenicol	0.062	25	7.6	8.2	^1^O_2_, •O_2_^−^	90% (120 min)	--	[132]
						12.6		100% (120 min)		
nZVI@CF	DO	MB	0.188	25	~9.5	6.68	•OH	96.8% (5 min)	--	[137]
		Brilliant green	0.124					96.4% (5 min)		
Pd/nZVI/rGO	DO	OTC	0.217	25	1.7	5.0	•OH	96.5% (60 min)	5	[141]
nZVI@C	DO	Nitrobenzene	0.325	25	4.6	7.05	•OH, •O_2_^−^	91% (120 min)	4	[142]
3D-rGO@nZVI/Al_2_O_3_	DO	Chloramphenicol	0.047	25	2.6	3.2	^1^O_2_, •O_2_^−^	100% (min)	--	[134]
						5.0		99% (min)		
						7.5		99% (min)		
						10.1		85% (min)		
nZVI/C	DO	Atrazine	0.046	25	1.1	3−9	•OH	93.5% (24 h)	4	[136]
A-nZVI	DO	Sb(III)	0.821	25	8.9	5.0	•OH	99% (30 min)	--	[138]
nZVI@D201	DO	SMX	0.14	25	~7.6	5.0	•OH	~98% (48 h)	--	[135]
nZVI/PA	DO	Ciprofloxacin	0.151	25	7.6	5.0	•OH, •O_2_^−^	98.5% (360 min)	5	[107]
3D-rGO@nZVI/MnO_2_	DO	OTC	0.109	25	--	3.0-6.5	•OH, ^1^O_2_	100% (120 min)	3	[143]
